# Highly accelerated phase-contrast MRI-based multi-directional flow imaging for peak velocity estimation in aortic stenosis patients.

**DOI:** 10.1186/1532-429X-18-S1-W22

**Published:** 2016-01-27

**Authors:** Juliana Serafim da Silveira, Adam V Rich, Yingmin Liu, Matthew Smyke, Ning Jin, Debbie Scandling, Jennifer A Dickerson, Carlos E Rochitte, Subha V Raman, Lee C Potter, Rizwan Ahmad, Orlando P Simonetti

**Affiliations:** 1 Dorothy M. Davis Heart and Lung Research Institute, The Ohio State University, Columbus, OH USA; 2 Department of Electrical and Computer Engineering, The Ohio State University, Columbus, OH USA; 3 College of Engineering, The Ohio State University, Columbus, OH USA; 4 Siemens Medical Solutions, Columbus, OH USA; 5 Department of Internal Medicine/Division of Cardiovascular Medicine, The Ohio State University, Columbus, OH USA; 6 Department of Medicine/Cardiology, InCor Heart Institute, São Paulo, Brazil; 7 Department of Radiology, The Ohio State University, Columbus, OH USA

## Background

Aortic stenosis (AS) is the most common valvular disease, and its prevalence is on the rise. Transthoracic echocardiography (TTE) is the current gold standard for diagnosis and grading of AS. However, TTE suffers from inadequate acoustic windows, and misalignment errors. While CMR has emerged as a robust tool for numerous applications, flow analysis by unidirectional phase-contrast MRI (PC-MRI) is known to underestimate velocity if the imaging plane is not set perpendicular to flow direction. Selecting the proper orientation can be challenging as the jet direction may vary with respect to the valve orifice. Thus, multi-directional flow imaging is likely to improve the accuracy of peak velocity (Vpeak) measurements. However, multi-directional acquisition can be prohibitively long, limiting its clinical utility. The purpose of this study is to apply a recently proposed data processing method called ReVEAL [1] to significantly accelerate multi-directional PC-MRI. ReVEAL exploits spatiotemporal sparsity and leverages the relationship between encoded and compensated images to enable highly accelerated PC-MRI.

## Methods

Patients with variable degrees of AS were prospectively enrolled and assessed with both TTE and ReVEAL. Three contiguous slices above the aortic valve were acquired with a 1.5T Siemens Avanto using the following parameters: TR/TE = 35.6/2.8 ms, α = 150, BW = 560 Hz/px, slice thickness = 8 mm, FOV = 280-360 mm, matrix =160 × 158, Venc = 150-450 cm/s, prospective triggering, and referenced 4-point encoding. A variable density sampling pattern [2] was used with a net acceleration rate of 8. Each slice was acquired in a 10s breath-hold. ReVEAL-based image recovery was performed on the three (x, y, z) encoding pairs. Reconstruction and analysis were performed offline using Matlab. Pixel-wise Vpeak was calculated as: Vpeak= √V_x_^2^ + V_y_^2^ + V_z_^2^. Magnitude and flow thresholds were applied to suppress noise pixels. Vpeak was defined as the maximum velocity within hand-drawn valve contours in all three slice planes. Vpeak from ReVEAL was then compared to clinically reported Vpeak by TTE.

## Results

**Figure 1:**
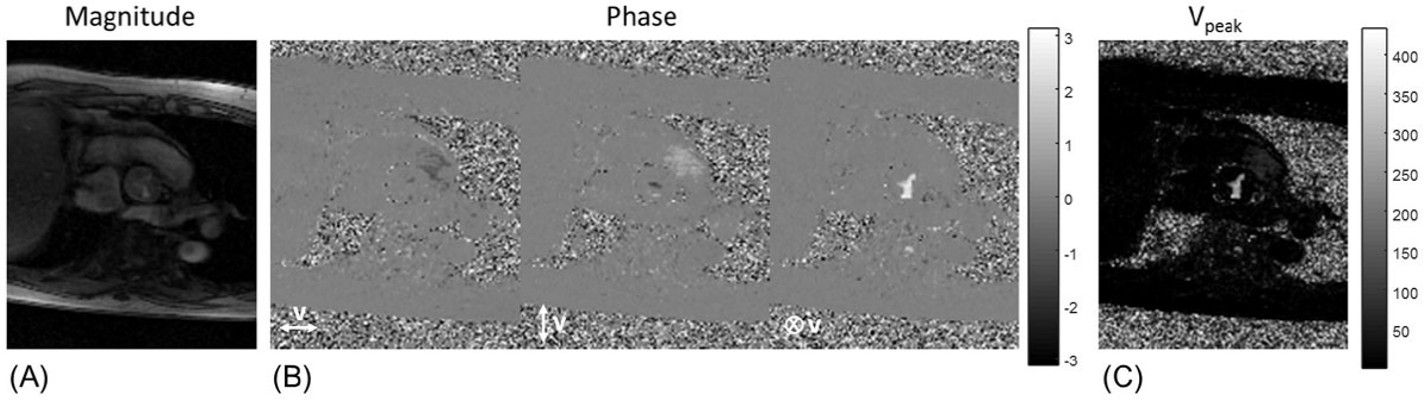
**Representative magnitude (A), phase (B) and Vpeak images (C) in a patient with mild aortic stenosis (peak velocity = 2.75 cm/s) using ReVEAL-based image recovery**.

**Figure 2:**
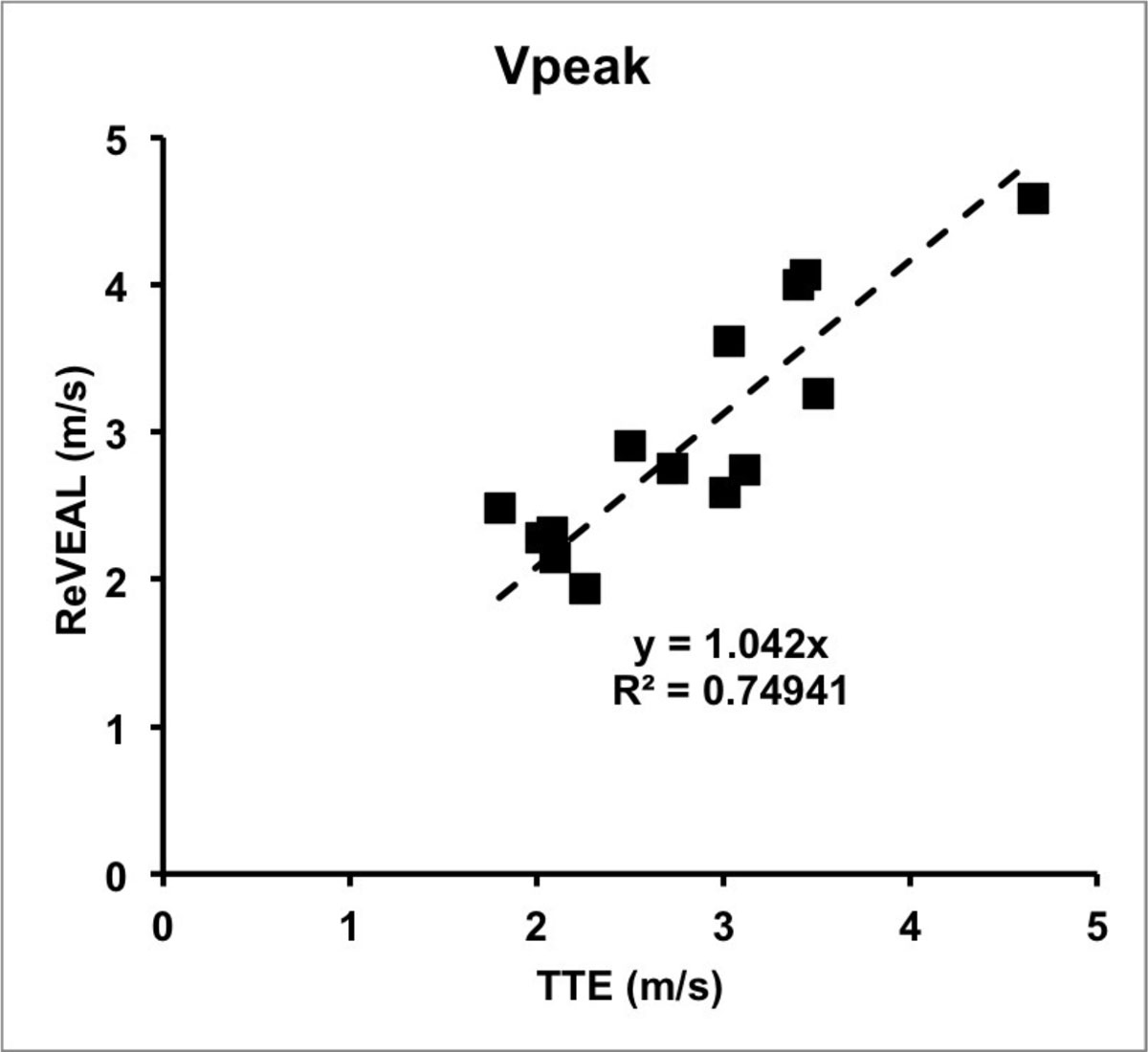
**Scatter plot of comparison between aortic peak velocities measured by ReVEAL and TTE**. A significant positive correlation was observed between both techniques.

Fourteen patients were included (7 males, median 68 years, range 27-82 years). Average interval between TTE and CMR was 40 days. Representative ReVEAL images are shown in figure 1. We found good correlation between ReVEAL and TTE (Figure 2), with an R^2^ = 0.75. In comparison to ReVEAL, TTE slightly underestimates Vpeak, which is not surprising as TTE is only sensitive to the flow that is parallel to the acoustic beam.

## Conclusions

While TTE can accurately measure velocity parallel to the acoustic beam, it is not sensitive to the other directions of flow. Therefore, multi-directional flow imaging, which encodes all three components of the velocity vector, can potentially outperform TTE in patients with eccentric or multiple jets. By exploiting structure unique to PC-MRI, ReVEAL enables multi-directional flow imaging in clinically feasible acquisition times.
